# *ST2L* Transmembrane Receptor Expression: An Immunochemical Study on Endarterectomy Samples

**DOI:** 10.1371/journal.pone.0156315

**Published:** 2016-05-25

**Authors:** Andrea Marzullo, Francesca Ambrosi, Mirjam Inchingolo, Fabio Manca, Fiorella Devito, Domenico Angiletta, Annapaola Zito, Pietro Scicchitano, Marco Matteo Ciccone

**Affiliations:** 1 Pathology Section, Department of Emergency and Organ Transplantation (DETO), Medical School, University of Bari, Bari, Italy; 2 Vascular Surgery Section, Medical School, Department of Emergency and Organ Transplantation (DETO), University of Bari, Bari, Italy; 3 Department of Science of Educational, Psychology and Communication-University of Bari, Bari, Italy; 4 Cardiovascular Diseases Section, Department of Emergency and Organ Transplantation (DETO), University of Bari, Bari, Italy; University of Kansas Medical Center, UNITED STATES

## Abstract

**Background:**

ST2 (suppression of tumorigenity) has been described as a receptor for the interleukin-33, a member of the IL-1 family of cytokines. It is associated to coronary artery disease, all-causes mortality and cardiovascular mortality.

**Aims:**

The present study was designed to assess the immunohistochemical expression of the ST2 receptor (ST2L/Il-1R) in atherosclerotic plaques of formalin fixed paraffin-embedded internal carotid arteries of patients with and without cerebro-vascular symptoms.

**Methods and Results:**

The study involved 41 cases (23 asymptomatic and 18 symptomatic). All the clinical and morphological parameters examined were uniformly distributed between the two groups, with a mild predominance of degree of calcification in asymptomatic cases (p = 0.01). ST2L expression was found to be more evident as a membrane pattern in macrophages when observing carotid atherosclerotic plaques of symptomatic patients, rather than in asymptomatic patients’ plaques (77.7% vs 39.1%; p = 0.015), and its expression was particularly remarkable in VI type plaque (AHA). Significantly, ST2L was marked by the endothelium of neoangiogenetic vessels on the shoulder region of the plaque, but not (apart from a few cases) in the endothelium covering the residual lumen of the vessel.

**Conclusions:**

The ST2L immunohistochemical expression was for the first time investigated in a large number of human carotid atherosclerotic plaques, as for its pattern of distribution in the different plaque cell populations. Furthermore, ST2L was particularly remarkable on macrophages, as a membrane pattern, of symptomatic patients’ plaque. Considering our data, we hypothesize that ST2L/IL33 axis could drive the mechanism of plaque development and eventually rupture.

## Introduction

Atherosclerosis is a diffuse and multisystem chronic inflammatory disease involving the vascular, metabolic and immune systems. It drives a progressive fatty deposit of lipid material, inflammatory cells, smooth muscle cells and extracellular matrix in the tunica intima [1–3].

The typical lesion is referred to *atheroma*. It is composed of a fibrous cap and extracellular lipids core. From a morphological and histological view point, atherosclerotic lesions can be differentiated into two categories: earlier or precursor (I-II-III types) and advanced lesions (IV-V-VI-VII-VIII types), according to American Heart Association classification [4–5]. Lesions considered advanced by their histology may or may not produce clinical manifestations [6]. Therefore, scientific community prefers pay more attention to the “*vulnerable plaque*” as the main determinant of atherothrombotic complications of atherosclerosis. The morphology and the inner composition of vulnerable plaque play a key role in the general prediction of events [7].

Several studies tried to identify the main features able to predict instability [8–10], although results are quite conflicting. For example, mechanical stresses from blood flow seem to enhance the instability of those plaques characterized by a soft lipid core, above all at the junction with the normal vessel (shoulder region) [11–12].

Recently, ST2 (suppression of tumorigenity) has been described as a receptor for the interleukin (IL)-33, a member of the IL-1 family of cytokines [13]. Its gene is located on chromosome 2q12 and transcripts for: IL1RL1-b or ST2L, which is a membrane receptor member of the IL-1 receptor family, and IL1RL1-a or sST2, the latter is a truncated soluble receptor that can be detected in serum [13]. sST2 is a decoy receptor for IL-33 and can be measured in the serum. Its serum levels are useful in risk stratification of patients suffering from myocardial infarction, heart failure and dyspnea [14–17]. According to current knowledge, IL-33 seems to be released during necrotic cell death, which is thought to be associated with tissue damage. For these properties, IL-33 was proposed to act as an “alarmin”. It binds ST2L on inflammatory cellular membranes, activating mitogen-activated protein kinase (MAPK) and several biochemical pathways [16]. ST2L is expressed on the surface of a wide variety of inflammatory cells: T helper 2 (Th2), mast cells, basophils, eosinophils [13–18]. The pathway ST2L/IL-33 drives the inflammatory response during asthma or atopic dermatitis [19]. Furthermore, it has been found that IL-33 amplifies the alternative activation of macrophages and other inflammatory cells [20]. In human endothelial cells, IL-33 induces inflammatory activation through up-regulation of IL-6, IL-8, monocyte chemoattractant protein-1 (MCP-1), vascular cell adhesion molecule-1 (VCAM-1), intercellular adhesion molecule-1 (ICAM-1), endothelial selectin (E-selectin), increases vascular permeability and promotes angiogenesis [21,22]. ST2L-IL-1RL1 effects on cardiovascular diseases and its role in atherosclerosis are less well known and still controversial. Even though *Miller et al*. [23] demonstrated that the IL-33-ST2L pathway might inhibit the development of atherosclerosis, recent studies revealed the association between IL-33-ST2L pathway and coronary artery disease [24], and the association of soluble ST2 levels with all-causes and cardiovascular mortality [25].

The aim of this study was to examine the expression of ST2L transmembrane receptor in human carotid samples and its distribution in the different cell populations that contribute to the formation of plaque atheroma (macrophages, monocytes, mast cells, lymphocytes and endothelial cells). Immunohistochemical results have been correlated with histological ones to understand how ST2L-IL1RL1 pathway could promote vulnerability of plaque and patients’ symptoms.

## Materials and Methods

### Study population

Atherosclerotic plaques were collected from 41 consecutive patients, between the age of 43 and 87 years old (mean 71.4) that underwent carotid endarterectomy (CEA) for internal carotid stenosis, in the Section of Vascular Surgery (Dept. of Emergency and Organ Transplantation) of University of Bari (see also S1 and S2 Tables).

Patients’ biographical, clinical and instrumental data were analyzed in order to identify cardiovascular risk factors and associated disorders.

Patients had been divided into two groups: symptomatic (23 patients—56%), defined according to guidelines of the Society of Vascular Surgery (SVS) [26,27], and asymptomatic (18 patients—44%). The definition of symptomatic is: a carotid extra-cranial lesion, which gives symptoms recognized as homolateral hemispheric ischemia and/or homolateral retinal ischemia. The symptoms have become apparent in the previous six months, and also in the absence of other embolic foci (atrial fibrillation, intracranial stenosis). The lesion is asymptomatic when it does not satisfy the previous definition. In case of the presence of homolateral hemispheric lesions shown by imaging methods (Computer Tomography or Nuclear Magnetic Resonance), that are symptomatic by definition.

All the plaques were observed at microscope by two observers in blind (AM, FA); the following histological features were considered: ulceration, intra-plaque hemorrage, micro-vascular density and the presence or not of a large, soft or necrotic core. At last, plaques were graded according to American Heart Association classification [4,5]. The study was approved by the Institutional Review Board of Bari Policlinic Hospital (IRB approval number 1413; October 26, 2009), University General Hospital and carried out in accordance with the principles of the Helsinki Declaration. All the patients gave their written consent to participate the study, the consents being kept in the department archives. The ethic committee/IRB approved this content procedure.

### Tissue Samples

After surgical dissection, plaques were fixed in 10% buffered neutral formalin for 24–48 hours, and cut into segments of 5 mm length. Afterwards, numbered sequentially to reconstruct the entire plaque in length from proximal common carotid artery to distal segment of internal carotid artery and included in paraffin blocks.

### Histology

Paraffin blocks were cut into 4 μm thick sections and stained with Hematoxylin-Eosin, Masson’s trichome and Van Gieson stains to evaluate the following histological parameters: (I) the degree of stenosis (estimated as > 70% or >90%); (II) the extension of lipidic core (estimated as absent, scarce estimated as less than 50% of the plaque area or large if more than 50% of the plaque was involved); (III) the inflammatory infiltration (estimated as absent, scarce if few inflammatory cells were detected or diffuse when cells were grouped in small clusters); (IV) the degree of calcification (estimated as absent, scarce in presence of isolated foci of calcification or diffuse when calcification involved large part of the plaque); (V) intraplaque hemorrhage (estimated as present or absent). In addition, estimation was made in regards to (VI) microvascular density in a semi quantitative way (absent, focal or diffuse).

### Immunohistochemistry

All plaques specimens were examined by immunohistochemistry for the expression of ST2L receptor in the whole section using a rabbit polyclonal anti-ST2 antibody (IL1RL1 interleukin receptor 1), (1:500 dilution; Sigma Aldrich). All immunostainings were done by an automated immunostainer (Dako Autostainer). Slices were pretreated using the DAKO-PT-link in a pH 9 EDTA retrieval solution. Substitution of the primary antibody with PBS served as a negative control. Colonic biopsies of patients affected by IBD were used as a positive control. Immunostainings for CD68 (clone PGM-1, 1:100 dilution, DAKO-Denmark), CD 45 (LCA, 1:500 dilution, DAKO-Denmark) and Tryptase (clone AA1, 1:150 dilution, DAKO) were also performed to evaluate the presence of inflammatory cells in the plaque, namely macrophages (CD 68 positive cells), lymphocytes (CD 45 positive cells) and mastocytes (tryptase positive cells).

### Statistics and data analysis

Data were collected and analyzed by SPSS.21Statistics. Clinical, histological and immunohistochemical results were compared between groups (*symptomatic* Vs *asymptomatic*) by **χ**^**2**^ test. A value of *p* <0.05 was considered statistically significant. Moreover, differences between the two groups and their associated procedures were analyzed using the statistical models of ANOVA.

## Results

The study of the 41 patients that underwent CEA was composed of 32 males (78%) and 9 females (22%), ranging from 43 to 87 years old (mean 71.4). According to the SVS guidelines [23,24], patients had to be divided into two groups: *asymptomatic* 23 (56.1%) and *symptomatic* 18 (43.9%).

There were no significant differences between the symptomatic and asymptomatic group in terms of age, sex, cardiovascular risk factors: hypertension (78% of asymptomatic patients Vs 67% of symptomatic patients), dyslipidemia (43% of asymptomatic patients Vs 50% of symptomatic patients) and diabetes (39% of asymptomatic patients Vs 33% of symptomatic patients). The studied population reflects a relatively typical population of patients with atherosclerotic disorder; 68.3% of our population had had other manifestations of vascular disease (ischemic heart attack, cerebral vascular occlusive disease, abdominal angina, intermittent claudication); (70% of asymptomatic patients Vs 67% of symptomatic patients).

Table 1 summarizes the cardiovascular risk factor results.

**Table 1:** Clinical characteristics of studied population.

*Cardiovascular risk factors*	*With symptoms*	*Without symptoms*	*P*
	***18 (43*.*9)***	***23 (56*.*1)***	
**Sex**			
M	16 (89)	16 (70)	0.135
F	2 (11)	7 (30)	0.515
**Age (years)**	67.6±10	74.4±8.5	0.438
**Hypertension**	12 (67)	18 (78)	0.316
**Hypercholesterolemia**	9 (50)	10 (43)	0.460
**Diabetes mellitus**	6 (33)	9(39)	0.479
**Vascular disease**	12 (67)	16 (70)	0.553
**COPD**	0 (0)	3 (13)	0.166
**Chronic Kidney Diseases**	0 (0)	2 (9)	0.309
**Atrial Fibrillation**	0 (0)	2 (9)	0.495
**Pts on statin therapy**	9 (50)	10 (43)	0.460

Number (percentages) of patients or mean±standard deviation; *p<0.05. COPD: Chronic Obstructive Pulmonary Disease.

**Fig 1:**
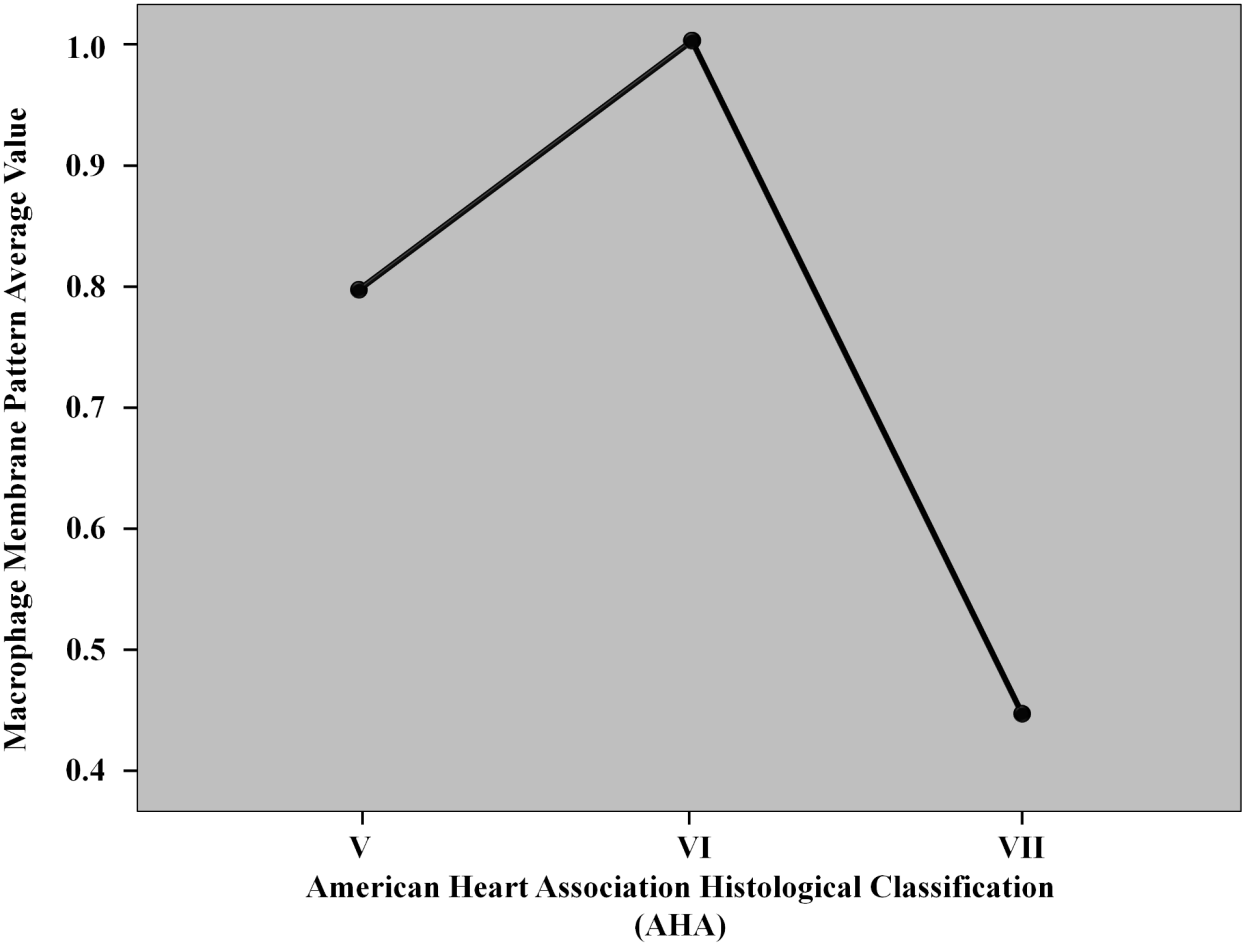
The graph illustrates the correlation between the histological classification (AHA^4-5^) and the expression of macrophage membrane pattern. The line chart witnessed an evident increasing of macrophage membrane pattern in VI class plaques. The histological classification of AHA defines VI class lesions as *atheroma*. Usually, type VI lesions episodes may quickly lead to occlusion and to be symptomatic for patients. It highlights our theories that the immunochemical ST2 membrane pattern has a direct correlation in terms of symptoms, and it could be a marker to detect vulnerable plaques.

Table 2 shows morphological plaque features and they were analyzed illustrating how it was not possible in our research to differentiate the two groups (*symptomatic Vs asymptomatic*). No statistically significant differences were found concerning: (I) degree of stenosis (>70% in 72.2% of symptomatic patients vs. 74% of asymptomatic patients; >90% in 27.8% of symptomatic patients vs. 26% of asymptomatic patients; *p =* 0.9035); (II) extension of the lipidic core (large in 39% of symptomatic patients vs. 65.2% of asymptomatic patients; scarce in 61% of symptomatic patients vs. 34.8% of asymptomatic patients; *p =* 0.086); (III) inflammatory infiltration (diffuse in 17.39% of asymptomatic patients Vs. 22.22% of symptomatic patients; scarce 82.61% of symptomatic patients Vs. 77.78% of symptomatic patients, *p =* 0.500); (IV) degree of calcification (diffuse in 34.8% of asymptomatic patients; scarce in 61.1% of symptomatic patients vs. 52.17% of asymptomatic patients; absent in 38.9% of symptomatic patients vs. 13.07% of asymptomatic patients; *p =* 0.01); (V) hemorrhage (present in 33.3% of symptomatic patients vs. 43% of asymptomatic patients; absent in 66.7% of symptomatic patients vs. 57% of asymptomatic patients; *p =* 0.621); (VI) micro-vascular density (absent in 22.2% of symptomatic patients vs. 26.1% of asymptomatic patients; focal in 33.3% of symptomatic patients vs. 21.8% of asymptomatic patients; diffuse in 44.5% of symptomatic patients vs. 52.1% of asymptomatic patients; *p =* 1.00). According to the American Heart Association classification, there were: plaque V type (27.8% of symptomatic patients); plaque VI type (11.1% of symptomatic patients vs. 13% of asymptomatic patients); plaque VII type (61.1% of symptomatic patients vs. 87% of asymptomatic patients) (Fig 1).

**Table 2:** Histological features of studied population.

*Hystology*	*With symptoms*	*Without symptoms*	*P*
	***18 (43*.*9)***	***23 (56*.*1)***	
**I Degree of stenosis**			
>70%	13 (72.2)	17 (74)	0.935
>90%	6 (27.8)	5 (26)	
**II Extention of lipidcore**			
Large	7 (39)	15 (65.2)	0.086
Scarce	8 (61)	8 (34.8)	
**III Inflammatory infiltration**			
Diffuse	4 (22.22)	4 (17.39)	0.500
Scarce	15 (71.78)	19 (82.61)	
**IV The degree of calcification**			
	---		
Diffuse	---	8 (34.8)	**0.01**
Scarce	11 (61.1)	12 (52.17)	
Absent	7 (38.9)	3 (13.07)	
**V Intraplaque Hemorrhage**			
Present	6 (33.3)	10 (43)	0.621
Absent	12 (66.7)	13 (57)	
**VI Microvascular density**			
Absent	4 (22.2)	6 (26.1)	1.000
Focal	6 (33.3)	5 (21.8)	
Diffuse	8 (44.5)	12 (52.1)	
**AHA**			
V	(27.8)	---	NS
VI	(11.1)	(13%)	
VII	(61.1)	(87%)	

Number (percentages) of patients; *p<0.05.

**Fig 2:**
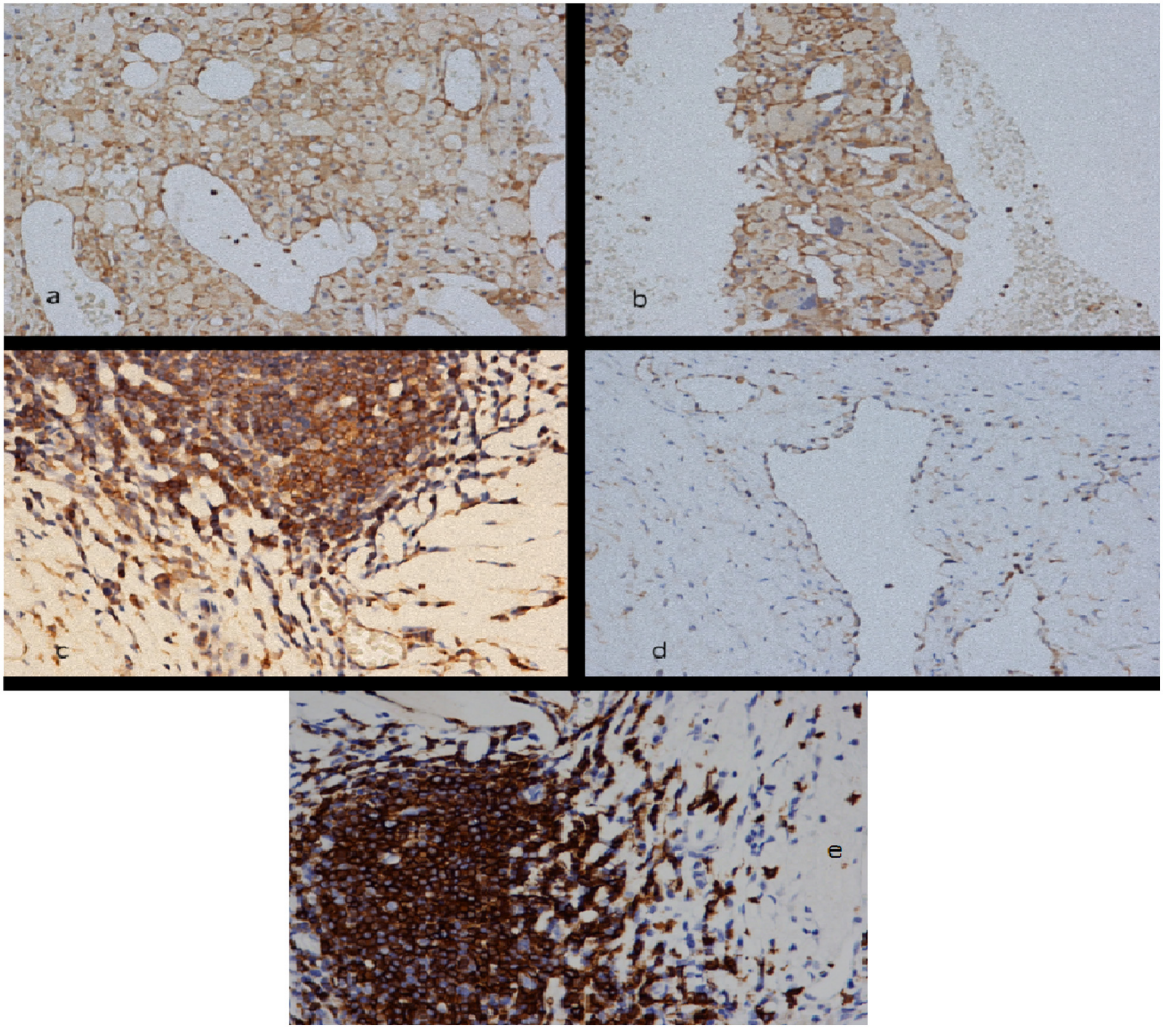
a). ST2 immuno-reactivity in macrophages with an evident membrane pattern. Concomitant expression in endothelial cells of new formed capillaries. (ST2 immunoreaction; 200 X original magnification). 2b) ST2 immuno-expression with a membrane pattern was particularly evident in foreign body giant cells of atherosclerotic plaques (ST2 immunoreaction; 200 X original magnification). 2c). A lymphatic follicle showing diffuse expression of ST2; a contiguous small capillary presents an intense endothelial positivity (ST2 immunoreaction; 400 X original magnification). 2d) Strong immuno-reactivity on endothelial cells of a newly formed vessel (ST 2 immunoreaction; 400 X original magnification). 2e) A lymphatic follicle (the same lymphatic follicle of Fig 2c) with intense immunostaining for CD 20; 400 X original magnification).

As depicted in Table 3, immunohistochemical analysis permitted the evaluation of the expression of ST2L transmembrane receptor in whole human carotid samples, in different cell populations. As shown in Fig 2d we detected ST2 receptor (ST2 o IL-1R1) in human carotid endarterectomy specimens on T-lymphocytes (confirmed by CD 3 immunostaining–Fig 2e), it was absent in 17.4% of asymptomatic patients vs. 11.1% of symptomatic patients; scarce/moderate in 82.6% of asymptomatic patients vs. 88.8% of symptomatic patients (*p =* 0.458); on macrophage cells with a membrane pattern, expressed in 39.1% of asymptomatic patients vs. 77.7% of symptomatic patients, (*p =* 0.015); on the luminal endothelium was 13.1% of asymptomatic patients vs. 16.6% of symptomatic patients (*p* = 0.642); on the endothelium of neoformed vessels was absent in 30.5% of asymptomatic patients vs. 16.6% of symptomatic patients; focal/diffuse in 69.9% of asymptomatic patients vs. 83.3% of symptomatic patients (*p =* 0.411). The other immunohistochemical markers, namely CD 68, CD 45 and tryptase were uniformly distributed in both populations of symptomatic and asymptomatic patients with no significant differences, apart from a light predominance (but also not statistically significant) of mastocytes in asymptomatic plaques in which a moderate amount of tryptase positive cells was found in 56.5% of cases respect to the 36.4% of symptomatic plaques. (see also S3, S4 and S5 Tables)

**Table 3:** Immuhistochemical results in the studied population.

*Immunohistochemistry ST2L (IL1R)*	*With symptoms*	*Without symptoms*	*P*
	***18 (43*.*9)***	***23 (56*.*1)***	
Mononuclear cells			
-	2 (11.1)	4 (17.4)	0.458
+/++	16 (88.8)	16 (82.6)	
Macrophage Membrane			
Pattern +	14 (77.7)	9 (39.1)	**0.015**
Lumen +	3 (16.6)	4 (13.1)	0.642
Endothelium of neoformed vessels			
-	4 (16.6)	7 (30.5)	0.411
++	14 (83.3)	16 (69.5)	

Number (percentages) of patients; *p<0.05.

## Discussion

The increased knowledge about sST2 and ST2L effects on cardiovascular system led physicians to consider the necessary assessment of plasma levels needed for sST2 as a novel biomarker of cardiovascular events and clinical conditions [14,15]. In the last few years, while researchers have focused on the significance of sST2 elevation in plasma, the significance of ST2L transmembrane receptor has received less investigation.

As atherosclerosis is a chronic inflammatory disorder, the endothelium plays a pivotal role in enhancing such a pathological process. According to *Demyanets et al*. [28], IL-33 could contribute to early events in endothelial activation by promoting adhesion molecules and pro-inflammatory cytokine expression in the endothelium. Endothelial cells express an array of adhesion molecules that controls events such as leukocyte rolling along and attaching to the endothelium and transmigration of leukocytes into areas of inflammation. This is considered a hallmark in the early pathogenesis of atherosclerosis. This group of researchers [28] for the first time showed that IL-33 induced rapid adhesion of leukocytes to monolayers of human endothelial cells isolated from coronaries and umbilical veins. The authors observed up-regulation in adhesion molecules (such as: VCAM-1, ICAM-1, E-selectin, and the chemokine MCP-1) production in these endothelial cells by means of IL-33 pathway. Such result confirmed the pro-atherosclerotic role of IL-33.

*Pollheimer et al*. [29] highlighted a new concept: quiescent endothelial cells are surprisingly resistant to pro-inflammatory activation by the allarmin IL-33, in comparison to the response induced by other pro-inflammatory cytokines (IL-1**β**, IL-4, interferon-**γ**, and tumor necrosis factor [TNF]-**α**). In particular, IL-33 selectively enhanced the expression of adhesion molecules and chemokines in non-quiescent, proliferating endothelial cells. This role is pro-atherosclerotic; in addition, proliferating endothelial cells are engaged in angiogenesis, which makes the plaque as unsTable. Furthermore, the inflammatory activation induced by IL-33 increases vascular permeability, inflammatory cytokines production, and stimulates angiogenesis [21,30].

It has also been shown that IL-33 induces the up-regulation of IL-6 and IL-8 in human endothelial cells [30] and Th2-dependent inflammatory diseases cytokines, such as IL-4 and IL-13, and enhances serum immune-globulin synthesis [13]. IL-33 acts as a promoter of Th2-dependent inflammatory disease and activates a number of cell types, including Th2 cells, mast cells and basophils. Therefore, IL-33 could inhibit the development of atherosclerosis in vivo by inducing a phenotypical Th1-to-Th2 switch, as atherosclerosis is considered as a Th1-driven chronic disease of the vasculature [31]. *McLaren et al*. [32] demonstrated that IL-33 could be a strong inhibitor of macrophage foam cells formation in vivo and in vitro, suggesting an atherosclerotic protective role. Furthermore, the expression of IL-33 and its receptor ST2 in murine and human cells and tissue reduces atherosclerosis development in ApoE^-/-^mice [33].

Nevertheless, other evidences showed the IL-33/ST2 pathway as able to promote atherosclerotic plaque development and instability, although sST2 levels have not been found elevated in patients that develop secondary cardiovascular events [34,35].

*Miller et al*. [23] observed that IL-33 was able to enhance the production of IL-4, -5 and -13 while decreasing the levels of interferon-γ. This means that the regulation of T-helper lymphocytes switches toward Th2 evolution rather than Th1 subtype, thus reducing the levels of chronic inflammation in vessels and the promotion of atherosclerosis [36, 37]. In atherosclerosis setting, lymphocytes T-helper seemed to show reduced levels of ST2 receptor and while a dysfunction in IL-33/ST2 axis was also able to dysregulate lymphocytes-Treg which usually reduce the inflammatory burden of systemic atherosclerosis [36]. Furthermore, *Miller et al*. [23] outlined the property of IL-33/ST2 axis in promoting the production of antibodies directed towards oxidized low-density lipoprotein: the theoretical consequence is the possible contrast of the development of cholesterol accumulation in vascular walls, i.e. a reduction in the early stage development of atherosclerosis.

Our study demonstrated that ST2L could have a key role in the pathogenesis of atherosclerotic plaque instability. In particular, we considered patients suffering from severe internal carotid stenosis who underwent CEA. They were differentiated into two groups: symptomatic and asymptomatic patients, according to SVS [26,27] guidelines.

According to histo-morphological features of plaques, our study did not show any significant differences between the two groups. Our data outlined that inflammatory infiltration, which can be considered as a marker for plaque instability, was developed in both groups. We observed that the degree of calcification was more pronounced in asymptomatic cases as compared to the symptomatic group, while the immunohistochemical expression of ST2L receptors was mostly outlined on macrophages of symptomatic patients’ plaques.

Therefore, we demonstrated for the first time the ST2L expression in the entire human carotid in formalin fixed paraffin embedded samples. We focused our attention on the pattern of expression in the different cell populations: mononuclear cells (lymphocytes), endothelium and macrophages. Mononuclear cells (identified as CD 3 positive T lymphocytes) strongly expressed ST2L in both symptomatic and asymptomatic plaques with no significant difference in term of distribution pattern.

Endothelium expression was also examined. Previous data showed some differences between the luminal endothelium and the neo-angiogenic one. In fact, endothelial cells covering the residual lumen of the vessel were less marked by ST2L receptor antibody as compared to the endothelium of the newly formed vessels. This confirms data reported in previous articles [28,29]: ST2L is a marker for non-quiescent endothelial cells and angiogenesis. Therefore, atherosclerotic plaques with large areas of neo-angiogenesis appear more prone to rupture. The observation that there were no semi-quantitatively differences in the density of plaque microvessels in both populations enforced our previous statement.

Our study, also, focused on the macrophage expression of ST2L: we found that these cells expressed the receptor with a membrane pattern. The membrane pattern of ST2L was strongly expressed if the macrophages infiltrating the shoulder region of the plaque had an epithelioid aspect. This pattern was more remarkable in symptomatic patients than asymptomatic ones: this seems to suggest its possible role in the pathogenetic mechanisms of atherosclerotic lesions progression and rupture. Although some articles support a protective role of IL33/ST2 pathway [32,33], the more evident expression of this macrophage membrane pattern in symptomatic plaques seems to correlate to symptoms due to plaque vulnerability.

## Limitations

Some limitations should be outlined. First of all, the small sample size can limit the validation of the results. Furthermore, we did not evaluate the soluble ST2 levels. Nevertheless, as the main purpose of this paper was to evaluate the role of ST2 in atherosclerotic plaques, the measurements of sST2 levels was not included in the initial study design. Future evaluations will certainly include the identifications of possible correlations between sST2 and ST2 distribution in carotid plaques.

Another limitation is related to the inclusion of data collected from a plaque while no information came from the entire artery. Nevertheless, the first attempt of our study was to firstly verify the possible distribution pattern of ST2 within the atherosclerotic plaques’ structure. The further evaluation of the distribution of such element within the remaining part of the artery will certainly be included in future research development. Finally, we did not consider a control tissue (as for example coming from healthy individuals with similar characteristics) as per ethic issues.

## Conclusions

In conclusion, for the first time we provide evidences about ST2L receptor expression in wide human cell coming from formalin fixed paraffin embedded carotid plaque samples. Such receptor was present both on T-cells and endothelial cells of neo-angiogenetic vessels, equally delivered in symptomatic and asymptomatic patients. Nevertheless, macrophages showed a membrane pattern of such a receptor, mostly represented in symptomatic patients’ atherosclerosclerotic plaques. We therefore hypothesized that the ST2/IL-33 pathway may play a central role in the novel mechanism that deserves further investigation in the role of vulnerability and plaque rupture.

## Supporting Information

S1 Table Characteristics of the study population and histological findings from the analysis of their carotid plaque. (DOC)

S2 Table Clinical characteristics of the study population. (DOC)

S3 Table Histological characteristics of the carotid plaques from symptomatic patients. (DOC)

S4 Table ST2 distribution on mononuclear/macrophages cells and on the endothelium (lumen/neoangiogenetic vessels). (DOC)

S5 Table Numerical data about distribution of ST2L on mononuclear/macrophages cells and on the endothelium (lumen/neoangiogenetic vessels). (DOC)
