# Supplementary material for: ST2L Transmembrane Receptor Expression: An Immunochemical Study on Endarterectomy Samples
Source: PLoS One. 2016 May 25;11(5):e0156315. doi: 10.1371/journal.pone.0156315 (PMC4880330; doi:10.1371/journal.pone.0156315)
Supplement: S1 Table — (DOC) [file pone.0156315.s001.doc]

**S1 table. Characteristics of the study population and histological findings from the analysis of their carotid plaque**

| **patient name** | **sex (1 M/ 0 F)** | **age** | **sympt/asympt (1 /0)** | **hypertension (yes 1/ no 0)** | **hypercolesterolemia (yes 1/no 0)** | **diabetes (yes 1/no 0)** | **vascular disease (yes 1/ no 0)** | **I Degree of stenosis** | **II extention of lipid core (0 scarce/1 evident)** | **III inflammatory infiltration (0 scarce/1 diffuse)** | **IV calcium (0absent/1 scarce/2 diffuse)** | **V Intraplaque Hemorrage (0 present/ 1 absent)** | **AHA** | **Mononuclear cells (- 0; +/++ 1)** | **Macrophage M.P. ( --- 0; + 1)** | **Lumen (--- 0; + 1)** | **Neoangiogenesis (- 0; +/++ 1)** |
| --- | --- | --- | --- | --- | --- | --- | --- | --- | --- | --- | --- | --- | --- | --- | --- | --- | --- |
| 1 | 0 | 58 | 0 | 0 | 0 | 0 | 1 | >70% | 0 | 1 | 1 | 1 | VII | 1 | 0 | 0 | 0 |
| 2 | 1 | 77 | 1 | 1 | 1 | 1 | 1 | >70% | 0 | 1 | 0 | 0 | VI | 1 | 1 | 1 | 0 |
| 3 | 1 | 74 | 0 | 1 | 1 | 0 | 1 | >70% | 0 | 0 | 1 | 1 | VII | 1 | 1 | 0 | 1 |
| 4 | 1 | 82 | 1 | 1 | 0 | 1 | 1 | >70% | 1 | 1 | 1 | 1 | VII | 0 | 1 | 0 | 1 |
| 5 | 1 | 74 | 0 | 1 | 1 | 1 | 1 | >70% | 0 | 0 | 2 | 1 | VII | 0 | 1 | 0 | 0 |
| 6 | 1 | 87 | 1 | 1 | 1 | 0 | 1 | >70% | 0 | 0 | 1 | 1 | VII | 1 | 1 | 0 | 1 |
| 7 | 1 | 85 | 0 | 1 | 0 | 1 | 1 | >90% | 1 | 0 | 1 | 0 | VII | 1 | 1 | 0 | 1 |
| 8 | 1 | 78 | 0 | 1 | 1 | 1 | 1 | >90% | 1 | 0 | 2 | 0 | VII | 1 | 0 | 0 | 1 |
| 9 | 0 | 77 | 0 | 1 | 0 | 0 | 1 | >70% | 1 | 0 | 2 | 1 | VII | 1 | 1 | 0 | 0 |
| 10 | 1 | 80 | 0 | 1 | 1 | 1 | 1 | >70% | 0 | 0 | 1 | 0 | VII | 1 | 0 | 0 | 1 |
| 11 | 1 | 83 | 0 | 1 | 0 | 0 | 1 | >90% | 1 | 0 | 2 | 0 | VI | 1 | 1 | 0 | 1 |
| 12 | 0 | 43 | 1 | 0 | 0 | 0 | 1 | >90% | 0 | 1 | 1 | 1 | VII | 1 | 1 | 0 | 1 |
| 13 | 1 | 82 | 0 | 0 | 0 | 0 | 0 | >70% | 1 | 0 | 1 | 1 | VII | 1 | 1 | 1 | 1 |
| 14 | 1 | 71 | 1 | 0 | 0 | 0 | 1 | >70% | 1 | 0 | 1 | 0 | VII | 0 | 1 | 1 | 0 |
| 15 | 0 | 72 | 0 | 1 | 1 | 0 | 0 | >70% | 0 | 0 | 1 | 1 | VII | 0 | 0 | 0 | 1 |
| 16 | 1 | 71 | 0 | 1 | 0 | 1 | 0 | >70% | 1 | 1 | 0 | 0 | VI | 1 | 1 | 0 | 1 |
| 17 | 1 | 64 | 1 | 1 | 1 | 0 | 0 | >70% | 0 | 0 | 1 | 0 | VII | 1 | 1 | 0 | 1 |
| 18 | 0 | 51 | 0 | 0 | 1 | 0 | 0 | >70% | 0 | 0 | 1 | 1 | VII | 1 | 0 | 1 | 1 |
| 19 | 1 | 70 | 1 | 1 | 0 | 0 | 1 | >90% | 0 | 1 | 0 | 1 | V | 1 | 1 | 1 | 0 |
| 20 | 1 | 66 | 0 | 1 | 1 | 1 | 1 | >90% | 1 | 0 | 1 | 0 | VII | 1 | 0 | 1 | 1 |
| 21 | 1 | 53 | 1 | 0 | 0 | 0 | 0 | >70% | 1 | 0 | 0 | 1 | V | 1 | 0 | 0 | 1 |
| 22 | 0 | 68 | 0 | 1 | 1 | 1 | 1 | >70% | 0 | 0 | 2 | 1 | VII | 1 | 0 | 0 | 1 |
| 23 | 1 | 59 | 0 | 1 | 0 | 1 | 1 | >90% | 1 | 0 | 0 | 0 | VII | 1 | 1 | 0 | 1 |
| 24 | 1 | 85 | 0 | 1 | 0 | 0 | 1 | >70% | 1 | 1 | 2 | 1 | VII | 1 | 0 | 0 | 0 |
| 25 | 1 | 77 | 0 | 1 | 1 | 0 | 1 | >70% | 0 | 0 | 2 | 0 | VII | 1 | 0 | 0 | 0 |
| 26 | 1 | 74 | 0 | 1 | 0 | 0 | 0 | >70% | 1 | 1 | 0 | 0 | VI | 1 | 1 | 0 | 1 |
| 27 | 1 | 61 | 1 | 1 | 1 | 1 | 1 | >70% | 1 | 0 | 1 | 1 | VII | 1 | 0 | 0 | 0 |
| 28 | 1 | 79 | 0 | 1 | 0 | 1 | 1 | >70% | 1 | 0 | 1 | 0 | VII | 1 | 0 | 0 | 0 |
| 29 | 1 | 66 | 1 | 0 | 0 | 0 | 0 | >70% | 1 | 0 | 1 | 0 | VII | 1 | 0 | 0 | 1 |
| 30 | 0 | 61 | 1 | 1 | 1 | 1 | 1 | >70% | 0 | 0 | 1 | 1 | VII | 1 | 0 | 0 | 1 |
| 31 | 1 | 76 | 0 | 1 | 0 | 0 | 1 | >70% | 1 | 0 | 1 | 1 | VII | 1 | 0 | 0 | 1 |
| 32 | 1 | 63 | 0 | 0 | 0 | 0 | 0 | >70% | 1 | 0 | 1 | 1 | VII | 1 | 0 | 0 | 1 |
| 33 | 0 | 77 | 0 | 1 | 1 | 0 | 1 | >90% | 1 | 0 | 2 | 1 | VII | 0 | 0 | 1 | 0 |
| 34 | 0 | 79 | 0 | 0 | 0 | 0 | 0 | >70% | 1 | 0 | 1 | 1 | VII | 0 | 0 | 0 | 1 |
| 35 | 1 | 76 | 1 | 1 | 0 | 1 | 1 | >70% | 0 | 0 | 0 | 1 | V | 1 | 1 | 0 | 1 |
| 36 | 1 | 76 | 1 | 1 | 1 | 0 | 0 | >90% | 1 | 0 | 0 | 1 | V | 1 | 1 | 0 | 1 |
| 37 | 1 | 74 | 1 | 1 | 1 | 1 | 1 | >70% | 1 | 0 | 1 | 1 | VII | 1 | 1 | 0 | 1 |
| 38 | 1 | 71 | 1 | 0 | 0 | 0 | 0 | >70% | 0 | 0 | 0 | 0 | VI | 1 | 1 | 0 | 1 |
| 39 | 1 | 73 | 1 | 1 | 1 | 0 | 1 | >70% | 0 | 0 | 1 | 1 | VII | 1 | 1 | 0 | 1 |
| 40 | 1 | 65 | 1 | 0 | 1 | 0 | 0 | >90% | 0 | 0 | 0 | 1 | V | 1 | 1 | 0 | 1 |
| 41 | 1 | 69 | 1 | 1 | 0 | 0 | 1 | >90% | 0 | 0 | 1 | 0 | VII | 1 | 1 | 0 | 1 |
