# Supplementary material for: ST2L Transmembrane Receptor Expression: An Immunochemical Study on Endarterectomy Samples
Source: PLoS One. 2016 May 25;11(5):e0156315. doi: 10.1371/journal.pone.0156315 (PMC4880330; doi:10.1371/journal.pone.0156315)
Supplement: S2 Table — (DOC) [file pone.0156315.s002.doc]

**S2 table. Clinical characteristics of the study population**

| **Patient** | **sex** | **age** | **asymp/sympt** | **hypertension** | **hypercolesterolemia** | **diabetes** | **vascular disease** | **other** |
| --- | --- | --- | --- | --- | --- | --- | --- | --- |
| 1 | F | 58 | asymp |  |  |  | yes |  |
| 2 | M | 77 | sympt | yes | yes | yes | yes | minor stroke, statin therapy |
| 3 | M | 74 | asymp | yes | yes |  | yes | chronic kidney disease, statin therapy |
| 4 | M | 82 | asymp | yes |  | yes | yes |  |
| 5 | M | 74 | asymp | yes | yes | yes | yes | statin therapy |
| 6 | M | 87 | sympt | yes | yes |  | yes | statin therapy |
| 7 | M | 85 | asymp | yes |  | yes | yes | atrial fibrillation, pulmonary arterial hypertension |
| 8 | M | 78 | asymp | yes | yes | yes | yes | colon diverticulosis, statin therapy |
| 9 | F | 77 | asymp | yes |  |  | yes |  |
| 10 | M | 80 | asymp | yes | yes | yes | yes | statin therapy |
| 11 | M | 83 | asymp | yes |  |  | yes | chronic obstructive pulmonary disease |
| 12 | F | 43 | sympt |  |  |  | yes |  |
| 13 | M | 82 | asymp |  |  |  |  |  |
| 14 | M | 71 | sympt |  |  |  | yes |  |
| 15 | F | 72 | asymp | yes | yes |  |  | chronic kidney disease, statin therapy |
| 16 | M | 71 | asymp | yes |  | yes |  | VII nerve paralysis |
| 17 | M | 64 | sympt | yes | yes |  |  | statin therapy |
| 18 | F | 51 | asymp |  | yes |  |  | statin therapy |
| 19 | M | 70 | sympt | yes |  |  | yes |  |
| 20 | M | 66 | asymp | yes | yes | yes | yes | statin therapy |
| 21 | M | 53 | sympt |  |  |  |  | amaurosis, upper limb deficiency |
| 22 | F | 68 | asymp | yes | yes | yes | yes | adrenal adenoma, statin therapy |
| 23 | M | 59 | sympt | yes |  | yes | yes | stroke |
| 24 | M | 85 | asymp | yes |  |  | yes |  |
| 25 | M | 77 | asymp | yes | yes |  | yes | chronic obstructive pulmonary disease, statin therapy |
| 26 | M | 74 | asymp | yes |  |  |  | chronic kidney disease, chronic lymphocytic leukemia, pulmonary nodule |
| 27 | M | 61 | sympt | yes | yes | yes | yes | statin therapy |
| 28 | M | 79 | asymp | yes |  | yes | yes | atrial fibrillation |
| 29 | M | 66 | sympt |  |  |  |  | minor stroke |
| 30 | F | 61 | sympt | yes | yes | yes | yes | statin therapy |
| 31 | M | 76 | asymp | yes |  |  | yes | chronic obstructive pulmonary disease |
| 32 | M | 63 | asymp |  |  |  |  |  |
| 33 | F | 77 | asymp | yes | yes |  | yes | statin therapy |
| 34 | F | 79 | asymp |  |  |  |  |  |
| 35 | M | 76 | sympt | yes |  | yes | yes |  |
| 36 | M | 76 | sympt | yes | yes |  |  | statin therapy |
| 37 | M | 74 | sympt | yes | yes | yes | yes | statin therapy |
| 38 | M | 71 | sympt |  |  |  |  |  |
| 39 | M | 73 | sympt | yes | yes |  | yes | chronic kidney diseases, statin therapy |
| 40 | M | 65 | sympt |  | yes |  |  | statin therapy |
| 41 | M | 69 | sympt | yes |  |  | yes |  |
