# Supplementary material for: ST2L Transmembrane Receptor Expression: An Immunochemical Study on Endarterectomy Samples
Source: PLoS One. 2016 May 25;11(5):e0156315. doi: 10.1371/journal.pone.0156315 (PMC4880330; doi:10.1371/journal.pone.0156315)
Supplement: S3 Table — (DOC) [file pone.0156315.s003.doc]

**S3 table. Histological characteristics of the carotid plaques from symptomatic patients.**

| **SYMPTOMATIC** | | | | | | |
| --- | --- | --- | --- | --- | --- | --- |
| **Pt** | **I degree of stenosis** | **II** | **III** | **IV** | **V** | **AHA** |
|  |  | **Extention of lipid core** | **Inflammatory** | **degree of calcification** | **Intraplaque** |  |
|  |  |  | **infiltration** |  | **hemorrage** |  |
| **2.** | >70% | scarce | diffuse | absent | present | VI |
| **4.** | >70% | evident | diffuse | scarce | absent | VII |
| **6.** | >70% | scarce | scarce | scarce | absent | VII |
| **12.** | >90% | scarce | diffuse | scarce | absent | VII |
| **14.** | >70% | evident | scarce | scarce | present | VII |
| **17.** | >70% | scarce | scarce | scarce | present | VII |
| **19.** | >90% | scarce | diffuse | absent | absent | V |
| **21.** | >70% | evident | scarce | absent | absent | V |
| **27.** | >70% | evident | scarce | scarce | absent | VII |
| **29.** | >70% | evident | scarce | scarce | present | VII |
| **30.** | >70% | scarce | scarce | scarce | absent | VII |
| **35.** | >70% | scarce | scarce | absent | absent | V |
| **36.** | >90% | evident | scarce | absent | absent | V |
| **37.** | >70% | evident | scarce | scarce | absent | VII |
| **38.** | >70% | scarce | scarce | absent | present | VI |
| **39.** | >70% | scarce | scarce | scarce | absent | VII |
| **40.** | >90% | scarce | scarce | absent | absent | V |
| **41.** | >90% | scarce | scarce | scarce | present | VII |
