# Supplementary material for: ST2L Transmembrane Receptor Expression: An Immunochemical Study on Endarterectomy Samples
Source: PLoS One. 2016 May 25;11(5):e0156315. doi: 10.1371/journal.pone.0156315 (PMC4880330; doi:10.1371/journal.pone.0156315)
Supplement: S4 Table — (DOC) [file pone.0156315.s004.doc]

**S4 table. ST2 distribution on mononuclear/macrophages cells and on the endothelium (lumen/neoangiogenetic vessels).**

|  | **ASYMTOMATIC** | | | | | **SYMTOMATIC** | | | | | |
| --- | --- | --- | --- | --- | --- | --- | --- | --- | --- | --- | --- |
|  | **ST2** | | | **ST2** | |  | **ST2L** | | | **ST2L endothelium** | |
|  | **inflammation** | | | **endothelium** | |  | **inflammation** | | |  |  |
| **Pt** | **Mononuclear cells** | **Macrophages** | | **Lumen** | **Neoangiogenesis** | **Pt** | **Mononuclear cells** | **Macrophages** | | **lumen** | **neoangiogenesis** |
|  |  |  |  |  |  |  |  |  |  |  |  |
|  |  |  | **Membrane Pattern** |  |  |  |  |  | **Membrane Pattern** |  |  |
| **1.** | **+** | **+** |  |  | **-** | **2.** | **++** | **++** | **+** | **+** | **-** |
| **3.** | **+** | **++** | **+** |  | **++** | **4.** | **-** | **+** | **+** |  | **+** |
| **5.** | **-** | **+** | **+** |  | **-** | **6.** | **+** | **++** | **+** |  | **+** |
| **7.** | **++** | **++** | **+** |  | **++** | **12.** | **++** | **+** | **+** |  | **+** |
| **8.** | **+** | **++** |  |  | **++** | **14.** | **-** | **+** | **+** | **+** | **-** |
| **9.** | **+** | **+** | **+** |  | **-** | **19.** | **++** | **++** | **+** | **+** | **-** |
| **10.** | **++** | **+** |  |  | **+** | **17.** | **++** | **++** | **+** |  | **+** |
| **11.** | **++** | **+** | **+** |  | **++** | **21.** | **++** | **++** |  |  | **++** |
| **13.** | **+** | **++** | **+** | **+** | **+** | **27.** | **+** | **-** |  |  | **-** |
| **15.** | **-** | **+** |  |  | **+** | **29.** | **+** | **++** |  |  | **+** |
| **16.** | **++** | **++** | **+** |  | **++** | **30.** | **+** | **+** |  |  | **+** |
| **18.** | **+** | **+** |  | **+** | **++** | **35.** | **++** | **++** | **+** |  | **+** |
| **20.** | **+** | **+** |  | **+** | **++** | **36.** | **++** | **++** | **+** |  | **+** |
| **22.** | **+** | **+** |  |  | **+** | **37.** | **+** | **+** | **+** |  | **+** |
| **23.** | **++** | **++** | **+** |  | **+** | **38.** | **++** | **++** | **+** |  | **++** |
| **24.** | **+** | **+++** |  |  | **-** | **39.** | **+** | **+** | **+** |  | **++** |
| **25.** | **+** | **+** |  |  | **-** | **40.** | **++** | **+++** | **+** |  | **++** |
| **26.** | **++** | **++** | **+** |  | **++** | **41.** | **++** | **+++** | **+** |  | **++** |
| **28.** | **+** | **++** |  |  | **-** |  |  |  |  |  |  |
| **31.** | **+** | **+** |  |  | **+** |  |  |  |  |  |  |
| **32.** | **+** | **+** |  |  | **+** |  |  |  |  |  |  |
| **33.** | **-** | **+** |  | **+** | **-** |  |  |  |  |  |  |
| **34.** | **-** | **+** |  |  | **+** |  |  |  |  |  |  |
|  |  |  |  |  |  |  |  |  |  |  |  |
|  |  |  |  |  |  |  |  |  |  |  |  |
| **-** | 4 |  |  |  | 7 |  | 2 | 1 |  |  | 4 |
| **+** | 13 | 14 | 9 | 4 | 8 |  | 6 | 6 | 14 | 3 | 9 |
| **++** | 6 | 8 |  |  | 8 |  | 10 | 9 |  |  | 5 |
| **+++** |  | 1 |  |  | 0 |  |  | 2 |  |  |  |
