# Supplementary material for: ST2L Transmembrane Receptor Expression: An Immunochemical Study on Endarterectomy Samples
Source: PLoS One. 2016 May 25;11(5):e0156315. doi: 10.1371/journal.pone.0156315 (PMC4880330; doi:10.1371/journal.pone.0156315)
Supplement: S5 Table — (DOC) [file pone.0156315.s005.doc]

**S5 table: numerical data about distribution of ST2L on mononuclear/macrophages cells and on the endothelium (lumen/neoangiogenetic vessels).**

| **ST2L** | | | | | | |
| --- | --- | --- | --- | --- | --- | --- |
|  | **MONONUCLEAR** | | **MACROPHAGE** | **LUMINAL** | **ENDOTHELIUM of NEOANGIOGENESIS** | |
|  | **CELLS** | | **MEMBRANE PATTERN** | **ENDOTHELIUM** |  |  |
|  | **-** | **+/++** |  | **+** | **-** | **+/++** |
| **Asymp.** | 4 | 19 | 9 | 4 | 7 | 16 |
| **23** | 17.39% | 82.61% | 39.13% | 17.39% | 30.43% | 69.57% |
| **Symp.** | 2 | 16 | 14 | 3 | 4 | 14 |
| **18** | 11.11% | 88.89% | 77.78% | 16.67% | 22.22% | 77.78% |
|  |  |  |  |  |  |  |
|  |  |  |  |  |  |  |
| **P** | 0.32 |  | 6.124 | 0.004 | 0.347 |  |
| **P yates** | 0.01 |  | 4.655 | 0 | 0.055 |  |
| **Odds ratio** | 0.59 |  | 5.44 | 0.95 | 0.65 |  |
